# Identification and Functional Validation of Autolysis—Associated Genes in *Lactobacillus bulgaricus* ATCC BAA-365

**DOI:** 10.3389/fmicb.2017.01367

**Published:** 2017-07-19

**Authors:** Xiaoyang Pang, Shuwen Zhang, Jing Lu, Lu Liu, Changlu Ma, Yang Yang, Panpan Ti, Weihua Gao, Jiaping Lv

**Affiliations:** ^1^Key Laboratory of Agro-Food Processing and Quality Control, Institute of Agro-Food Science and Technology, Chinese Academy of Agricultural Science Beijing, China; ^2^Beijing Advanced Innovation Center for Food Nutrition and Human Health, Beijing Technology and Business University Beijing, China; ^3^Department of Food and Biological Engineering, Beijing Vocational College of Agriculture Beijing, China

**Keywords:** two component system, autolysis, *Lactobacillus delbrueckii*, lactic acid bacteria, gene knockout

## Abstract

Lactic acid bacteria (LAB) are important organisms in food production. Indeed, LAB autolysis is very critical in dairy processing. For example, it influences the development of cheese flavor by releasing intracellular enzymes, and controls cell growth in yogurts and probiotic products. Two component systems (TCS) constitute essential environmental sensors and effectors of signal transduction in most bacteria. In the present work, mutants of one TCS (LBUL_RS00115/LBUL_RS00110) were generated to assess the relationship between TCS and cell autolysis. The mutants displayed decreased autolysis in comparison with wild type; meanwhile, complementation reversed this effect. The interaction between LBUL_RS00115 and LBUL_RS00110 was confirmed by yeast two-hybrid analysis. These observations suggested that the TCS (LBUL_RS00115/LBUL_RS00110) was involved in autolysis in *Lactobacillus delbrueckii* subsp. *bulgaricus*.

## Introduction

Lactic acid bacteria (LAB) are common starters for the production of yogurt and other dairy products, and their autolysis attracts increasing attention (Cibik and Chapot-Chartier, 2000; Ouzari et al., 2002; Ortakci et al., 2015). During the process of cheese production, ripening is critical for its role in determining final product flavor and texture, which constitute the basis for cheese product differentiation (Lazzi et al., 2016). This lengthy procedure (from about 3 weeks to >2 years) renders cheese production costly (Sondergaard et al., 2015). Attempts to speed up ripening include temperature increase (Fox et al., 1996), starter culture adjustment (Williams et al., 2006; Garbowska et al., 2015), and enzyme supplementation (Fox et al., 1996). Starter strain lysis in the ripening step releases cytoplasmic enzymes that degrade amino acids into cheese (Xu and Kong, 2013). Such enzymes are believed to promote the degradation of peptides, also removing the bitter ones (Valence et al., 2000; Collins et al., 2003; Visweswaran et al., 2017). It is therefore important to induce LAB starter autolysis during cheese production. While production yogurts, LAB autolysis lowers live cell count of starters (Pang et al., 2014). This demonstrates the significant role of LAB autolysis in food production, and unveiling the underlying mechanisms is of prime importance.

Bacteria usually sense and react to various changes in their environment via two component systems (TCS) (Cui et al., 2012). TCS are found in most bacteria, primarily as essential environmental sensors and cell signaling effectors (El-Sharoud, 2005; Thevenard et al., 2011; Zuniga et al., 2011). TCS typically consist of a membrane-bound histidine protein kinase (HPK) (sensor) and a soluble response regulator (RR, signaling effector) (El-Sharoud, 2005; Thevenard et al., 2011; Zuniga et al., 2011; Borland et al., 2015).

Genome sequencing predicts multiple TCS in LAB (Thevenard et al., 2011), with many remaining uncharacterized. TCS were shown to be associated with the production of bacteriocins (Roces et al., 2012; Marx et al., 2014). The TCS LBA1524/LBA1525 of *L. acidophilus* is implicated in acid tolerance (Azcarate-Peril et al., 2005), and LBA1430/LBA1431 in bile tolerance (Pfeiler et al., 2007). The TCS lamBDCA system of *Lactobacillus plantarum* is likely to affect commensal host–microbe interactions, since a lamA mutant adheres to surfaces (Sturme et al., 2005).

Autolysis of LAB usually occurs at high cell density (Chu et al., 2013; Kovacs et al., 2013; Hong et al., 2014), TCS enable bacteria to sense, respond, and adapt to a wide range of environments, stressors, and growth conditions (Faralla et al., 2014; Straube, 2014; Yu et al., 2014). It has not been confirmed whether there is a correlation between cell autolysis and TCS. The current work aimed to assess the association of TCS and *L. bulgaricus* cell autolysis by gene knockout techniques. Our findings would provide a strong basis for directional regulation of LAB autolysis.

## Materials and methods

### Bacterial cultures

Table 1 lists all strains and plasmids utilized. *E. coli* and *L. bulgaricus* were cultured in LB and Man-Rogosa-Sharpe (MRS) (Beijing Land Bridge Technology Co., Ltd. CM187), respectively, at 37°C with no shaking. *Saccharomyces cerevisiae* Y_2_HGOLD cells, carrying four reporter genes (*HIS3, ADE2, AUR1-C*, and *MEL1*) controlled by the GAL4 promoter (Xu et al., 2015), were cultured in Yeast Peptone Dextrose (10 g yeast extract, 20 g peptone, 20 g dextrose per liter) or synthetic defined (SD) medium (BD Difco Ltd., USA) at 28°C. Ampicillin (Amp, Sigma Chemical Co, USA), was used for *E. coli* at 100 μg/mL. Erythromycin (Em, Sigma) and Amp were used for *L. bulgaricus* at 50 μg/mL each. Chloramphenicol was used for *L. bulgaricus* at 10 μg/mL.

**Table 1:** Bacterial strains and plasmids used in this study.

**Strain or plasmid**	**Relevant genotype or description**	**Reference and/or source**
Strains *E. coli* DH5α	F^−^, φ80d *lac*Z ΔM15, Δ(*lacZYA*-*argF*)U169, *deoR, recA*1, *endA*1, *hsdR*17 (rk^−^,mk^+^), *phoA, supE*44, λ^−^, *thi*-1, *gyrA*96, *relA*1	TaKaRa
*L. bulgaricus* ATCC BAA-365	Wild-type *L. bulgaricus*	ATCC
*L. bulgaricus* Δ*H4160-1*	LBUL_RS04160 gene mutant of *L. bulgaricus* ATCC BAA-365; *HPK4160::EryB*	This study
*L. bulgaricus* Δ*H0115-1*	LBUL_RS00115 gene mutant of *L. bulgaricus* ATCC BAA-365; *HPK0115::EryB*	This study
*L. bulgaricus rH0115-1*	Complementation of the LBUL_RS00115 mutant with pMG56e carrying LBUL_RS00115 gene	This study
*S. cerevisiae* Y_2_HGOLD	the HIS3, ADE2, and MEL1/AUR1-C reporter genes are under the control of Gal4-responsive promoter elements-G1, G2, and M1	Clontech
Plasmids		
pMD18T	clone vector; Amp^r^	TaKaRa
pUC19	clone vector; Amp^r^	TaKaRa
pMG76e	Expression vector of lactic acid bacteria; Em^r^	College of food science and Nutritional Engineering, China Agricultural University
pMG56e	Expression vector of lactic acid bacteria; Cm^r^, derivative of pMG36e in which the gene coding for erythromycin resistance was replaced with a gene coding for chloramphenicol resistance from pNZ8148	College of food science and Nutritional Engineering, China Agricultural University
pUC19-HPK4160	pUC19 derived integration vector containing the LBUL RS04160 gene with S*ph*I, *EcoR*I restriction enzyme sites; Amp^r^	This study
pUC19-HPK4160::EryBII	pUC19-HPK4160 derived integration vector containing the EryBII gene; Em^r^; Amp^r^	This study
pUC19-HPK0115	pUC19 derived integration vector containing the LBUL RS00115 gene with S*ph*I, *EcoR*I restriction enzyme sites; Amp^r^	This study
pUC19-HPK0115::EryBII	pUC19-HPK0115 derived integration vector containing the EryBII gene; Em^r^; Amp^r^	This study
pMG56e-HPK0115	pMG56e derived expression vector carrying full LBUL_RS00115 gene; Cm^*r*^	This study
pGBKT7	Plasmid in yeast two-hybrid system; Gal4 (1–147), Trp1, Kan^r^	Clontech
pGADT7	Plasmid in yeast two-hybrid system; Gal4 (768–881), Leu2, Amp^r^	Clontech
pGBKT7-53	Positive control plasmid that encodes a fusion of the murine p53protein (72–390) and the GAL4 DNA-BD (1–147)	Clontech
pGBKT7-Lam	Negative control plasmid that encodes the Gal4 BD fused with lamin	Clontech
pGADT7-T	Positive control plasmid that encodes a fusion of the SV40 large T antigen (87–708) and the GAL4 AD (768–881)	Clontech
pGBK-HPK0115	pGBKT7 derived expression vector carrying full LBUL_RS00115 gene; Kan^r^	This study
pGAD-RR0110	pGADT7 derived expression vector carrying full LBUL_RS00110 gene; Amp^r^	This study

### Prediction of two component system in *L. bulgaricus*

The whole genome sequence of the *L. bulgaricus* ATCC BAA-365 strain was downloaded from NCBI (https://www.ncbi.nlm.nih.gov/nuccore/NC_008529.1), and used for the prediction of TCS. HisKA (PF00512), HATPase-c (PF02518), Response reg (PF00072) from the Protein families database were used in a HMMER search. HisKA.hmm and HATPase-c.hmm were employed for scanning the highly conserved phosphate group binding- and HATPase regions in HPK. Response-reg.hmm was utilized to screen conserved phosphate groups in the response regulator protein.

### Construction of recombinant plasmids

**Figure 1:**
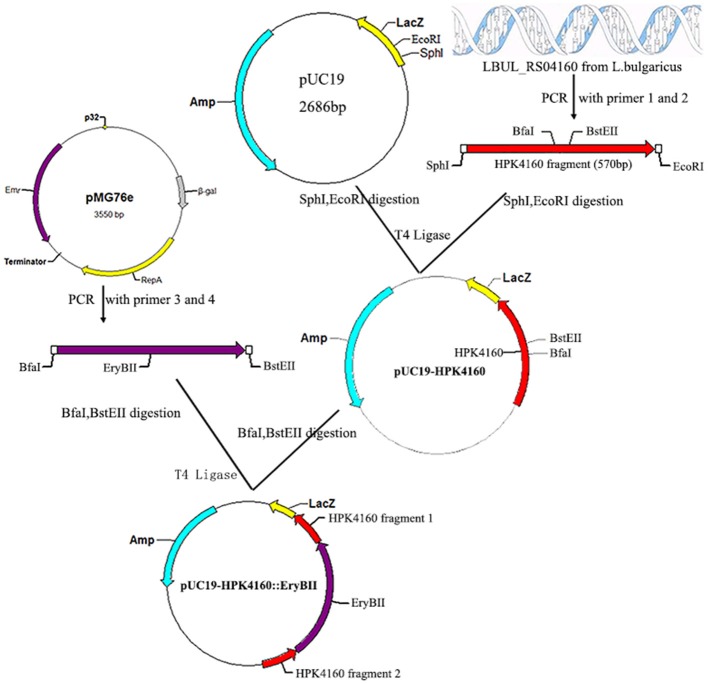
Construction of recombinant plasmid pUC19-HPK4160::EryBII. The red arrow represents the HPK4160 gene from *L. bulgaricus* ATCC BAA-365; The cyan arrow represents the ampicillin resistance gene; The purple arrow represents the erythromycin resistance gene; SphI, EcorI, BfaI, BstEII are four endonuclease hydrolysis sites.

To construct a LBUL_RS04160 mutant, a 570 bp fragment intermediate region of the LBUL_RS04160 gene was first amplified by PCR using HPK4160-SphI-F (5′-ACGCGCATGCCGCATGAACTTAAGACGCCC-3′) (SphI site underlined) and HPK4160-EcoRI-R (5′-ACATGAATTCTTGCGGCTGTGGCTCTTATC-3′) (EcoRI site underlined) primers, and inserted into pUC19 to generate pUC19-HPK4160. The erythromycin resistance gene was amplified by PCR from pMG76e using the EryB-BfaI-F (5′-CCGCTAGATGACCACCGACGCCGCGACG-3′) and EryB-BstEII-R (5′-CGGGTAACCTCACTGCAACCAGGCTTCCGG-3′) primers, and inserted into pUC19-HPK4160 to create plasmid pUC19-HPK4160::EryBII (Figure 1).

**Figure 2:**
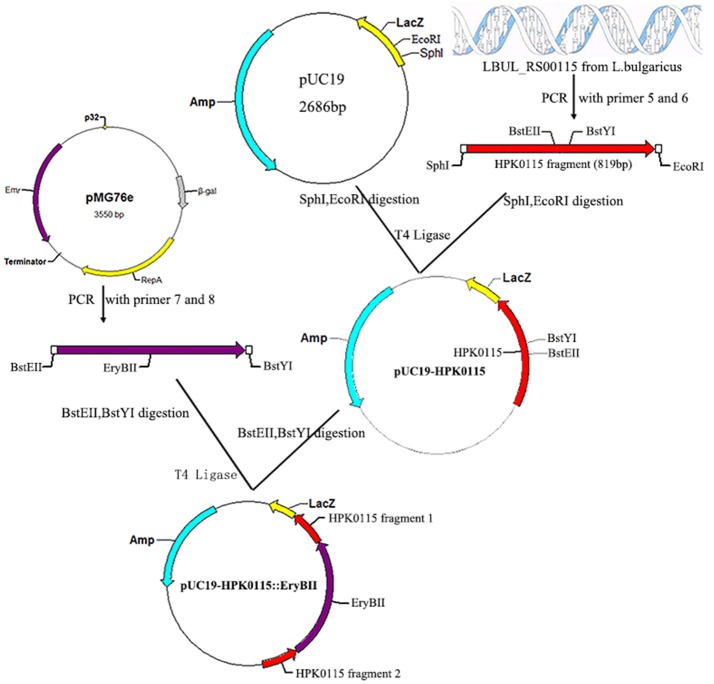
Construction of recombinant plasmid pUC19-HPK0115::EryBII. The red arrow represents the HPK0115 gene fragment from *L. bulgaricus* ATCC BAA-365; The cyan arrow represents the ampicillin resistance gene; The purple arrow represents the erythromycin resistance gene; SphI, EcorI, BstEII, BstYI are four endonuclease hydrolysis sites.

To construct a LBUL_RS00115 mutant, a 819 bp fragment intermediate region of the LBUL_RS00115 gene was first amplified by PCR with the HPK0115-SphI-F (5′-ACGCGCATGCCGCGGGCAGGCAAAAAG-3′) (SphI site underlined) and HPK0115-EcoRI-R (5′-ACATGAATTCAACGCAGCGGATGATGCTTA-3′) (EcoRI site underlined) primers, and inserted into pUC19 to generate pUC19-HPK0115. The erythromycin resistance gene was amplified by PCR from pMG76e with the EryB-BstEII-F (5′-CGGGTAACCATGACCACCGACGCCGCGACG-3′) and EryB-BstYI-R (5′-CGAGATCCTCACTGCAACCAGGCTTCCGG-3′) primers, and inserted into pUC19-HPK0115 to create the pUC19-HPK0115::EryBII plasmid (Figure 2).

All other gene manipulation experiments were carried out as described previously (Pang et al., 2014).

### Transformation of *L. bulgaricus*

The pUC19-HPK4160::EryBII and pUC19-HPK0115::EryBII plasmids were transformed into *L. bulgaricus* ATCC BAA-365 as proposed previously (Holo and Nes, 1989; Kim et al., 2009), with minor modifications described in a previous study (Pang et al., 2014).

### Complementation of mutants by the *L. bulgaricus* LBUL_RS00115 gene

A 1.3 kb fragment, encompassing the complete LBUL_RS00115 coding region was amplified by PCR using the HPK0115-SalI-F (5′-ACGCGTCGACATGATCAACAGCCTGTTCA-3′) and HPK0115-SphI-R (5′-ACATGCATGCCTATCCCTTCTGAATAACT-3′) primers from the *L. bulgaricus* BAA-365 genome, and cloned into pMG56e to generate pMG56e-HPK0115. The latter plasmid (pMG56e-HPK0115) was then introduced into the mutant *L. bulgaricus H0115-K7*, yielding complemented strain *L. bulgaricus H0115-K7-com1* (Table 1). Transformants with successful complementation were selected by culture on 10 μg/mL chloromycetin containing plates.

### Autolysis assessment in LAB

Bacterial suspension (OD600 = 0.4~0.6) was centrifuged to remove the bacterial cells, the supernatant was measured OD260/280 nm, the reading was recorded as A_0_. Take the appropriate amount of the above bacterial suspension in the incubator for t hours. Half of the sample was centrifuged to remove the bacterial cells. Measure the OD260/280 nm of the supernatant and the reading was recorded as A_t_. The remaining bacterial suspension sonicated (400 w, work 3 s, interval 3 s) until the solution became clear (the cells are completely broken) under ice-cooling, bacterial cells were removed by centrifugation, measure the OD260/280 nm of the supernatant and the reading was recorded as A_s_. The autolysis rate is calculated according to the formula:

(1) $$\text{Autolysis rate}/\%=({\text{A}}_{\text{t}}-{\text{A}}_{\text{0}})/({\text{A}}_{\text{s}}-{\text{A}}_{\text{0}})*\text{100}\%$$

Groups were compared by One-Way ANOVA and LSD test.

### Yeast two-hybrid analysis between WP_011677872.1 and WP_011677871.1

**Figure 3:**
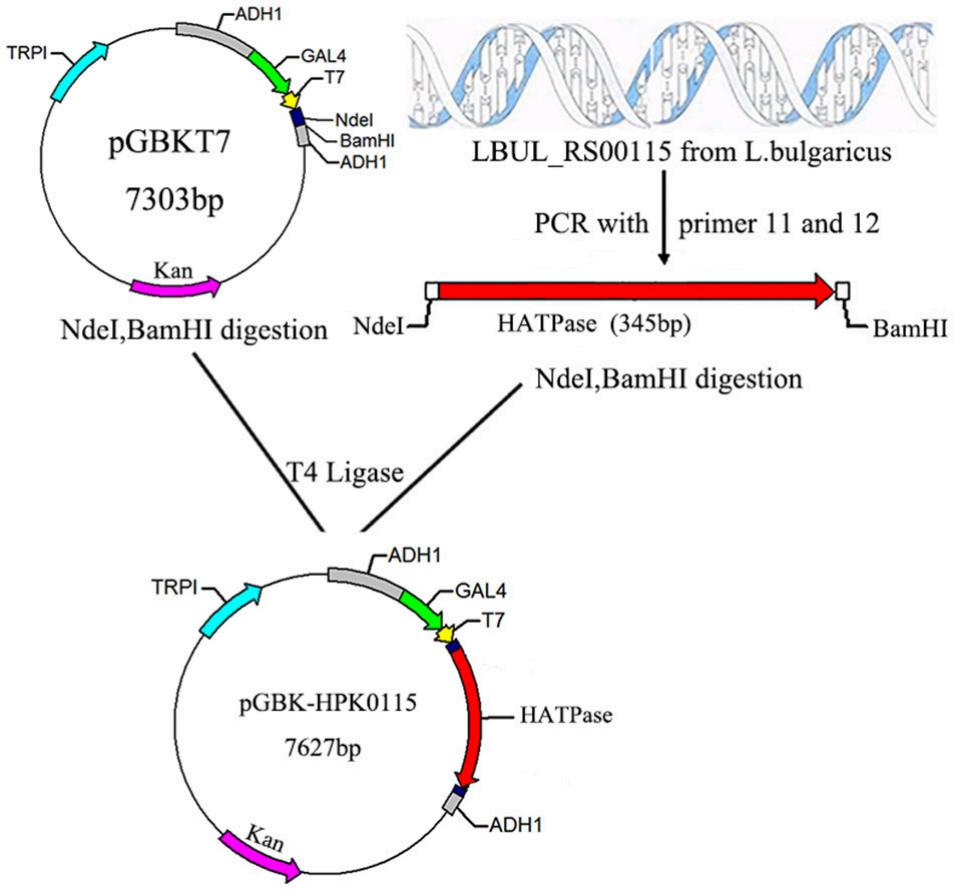
Construction of recombinant plasmid pGBK-HPK0115. The red arrow represents the LBUL_RS00115 gene fragment from *L. bulgaricus* ATCC BAA-365; The magenta arrow represents the kanamycin resistance gene; NdeI, BamHI are endonuclease hydrolysis sites.

**Figure 4:**
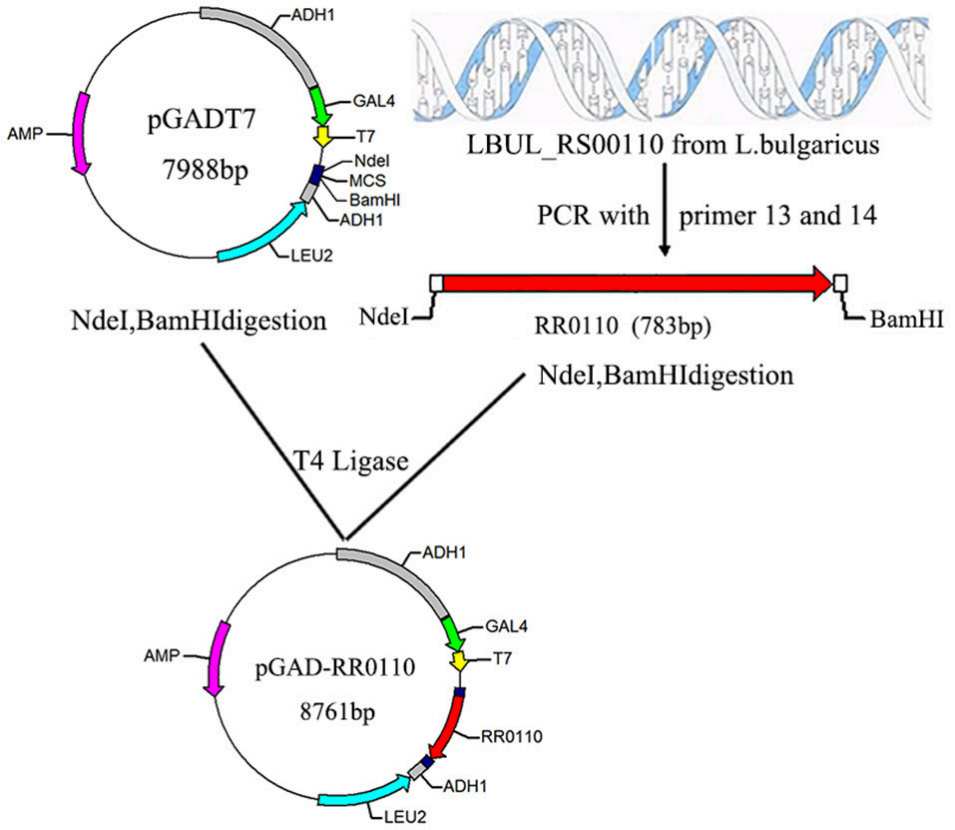
Construction of recombinant plasmid pGAD-RR0110. The red arrow represents the LBUL_RS00110 gene fragment from *L. bulgaricus* ATCC BAA-365; The magenta arrow represents the ampicillin resistance gene; NdeI, BamHI are endonuclease hydrolysis sites.

It is difficult to construct a two hybrid system by using full length WP_011677872.1 and WP_011677871.1 genes, because WP_011677872.1 contain six transmembrane regions. Therefore, these transmembrane regions of WP_011677872.1 were removed, and the HATPase-c domain of WP_011677872.1 was selected as the bait protein. The WP_011677872.1 HATPase-c domain gene was PCR amplified with the HATPase-NdeI-F (5′-GGAATTCCATATGATGGTAAATATCGTAAGCATCA-3′) and HATPase-BamHI-R (5′-CGGGATCCCTATCCCTTCTGAATAACT-3′) primers, and inserted into the pGBKT7 vector (Clontech), to create the two-hybrid plasmid pGBK-HPK0115 (Figure 3); the complete LBUL_RS00110 sequence was amplified by PCR using the RR0110- NdeI-F (5′-GGAATTCCATATGATGCTAGCCATCATCATTT-3′) and RR0110-BamHI-R (5′-CGGGATCCTTAAACAAGGTCATTTT-3′) primers, and inserted into the pGADT7 vector (Clontech), to create the two-hybrid plasmid pGAD-RR0110 (Figure 4).

Co-transformation of *S. cerevisiae* Y_2_HGOLD was carried out using various two-hybrid plasmid pairs: pGBK-HPK0115/pGAD-RR0110 (0.15 μg/each), pGBKT7-53/pGADT7-T (positive control), pGBKT7-Lam/ pGADT7-T (negative control). Y_2_HGold cell amounts were determined with (His) or without (-His) histidine, to assess HIS3 reporter activation, which in combination with AUR1-C reporter activation (growth on 0.1 mg/mL aureobasidin A, Clontech), reflects protein interaction. Strain *S. cerevisiae* Y_2_HGold, and various vectors were obtained from Clontech Laboratories, Inc.

## Results

### TCS distribution in *L. bulgaricus* BAA-365

**Figure 5:**
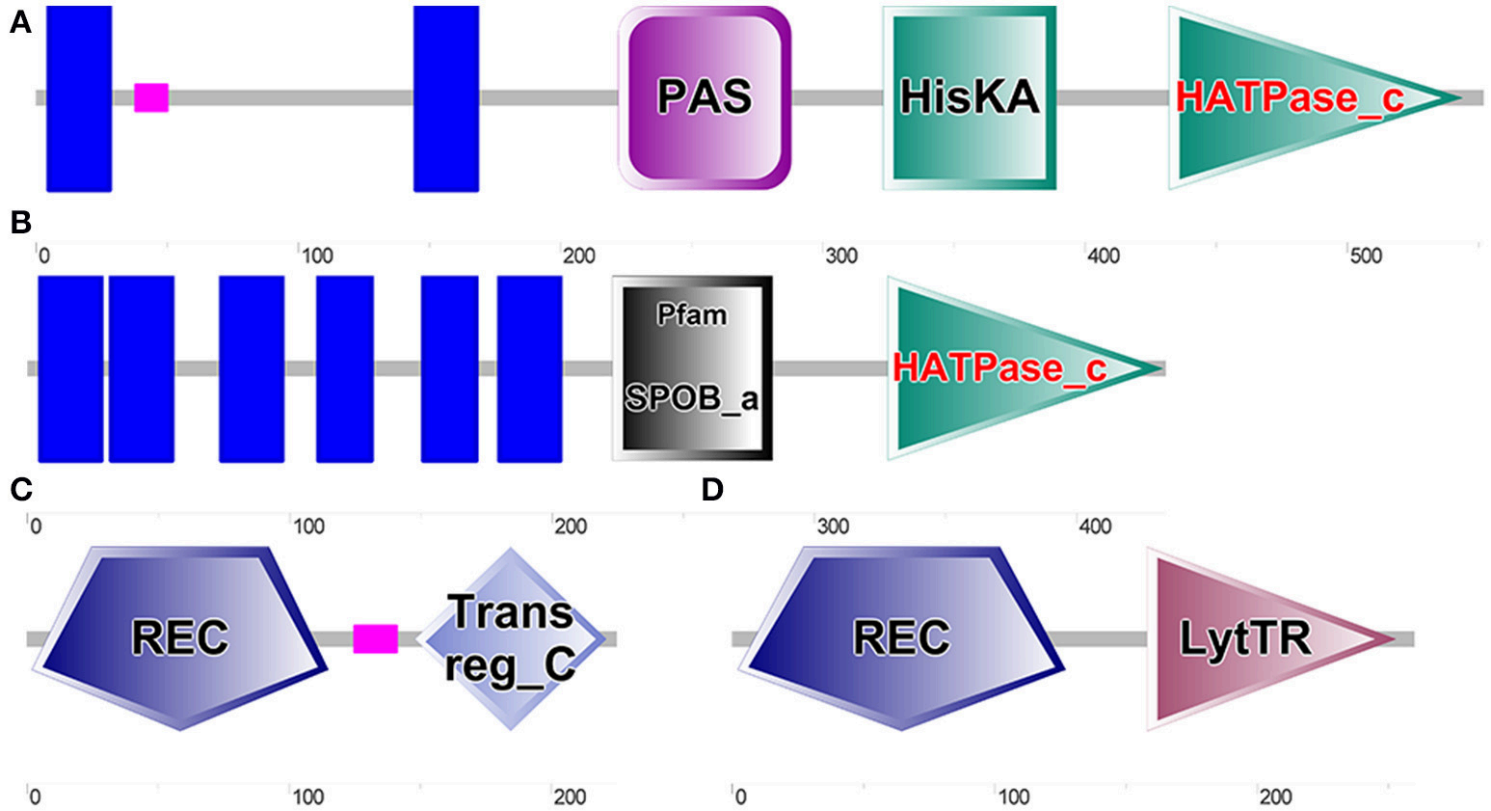
Results of SMART analysis of four proteins. **(A)**. WP_011543855.1 (Encoded by LBUL_RS04160, CDS Region in Nucleotide: 811449-813107); **(B)** WP_011677872.1 (Encoded by LBUL_RS00115, CDS Region in Nucleotide: 23723-25027); **(C)** WP_003620064.1 (Encoded by LBUL_RS04155, CDS Region in Nucleotide: 810772-811448); **(D)** WP_011677871.1 (Encoded by LBUL_RS00110, CDS Region in Nucleotide: 22935-23717).

The whole genome sequence of *L. bulgaricus* BAA-365 was scanned by the Hmmer software for HisKA, HATPase-c and Response-reg domains. A total of 7 HPKs and 7 RRs were identified, as shown in Table 2. NCBI BLASTP was used for HPK and RR function prediction: the functions of five RRs have been reported, while those of the two remaining RRs remain unknown. The structural domains of WP_011543855.1, WP_003620064.1, WP_011677872.1, and WP_011677871.1 were assessed by utilizing Simple Modular Architecture Research Tool (SMART) (Figure 5).

**Table 2:** Functional prediction of *L. bulgaricus* BAA-365 TCS system.

**HK protein no**.	**RR protein no**.	**HK/RR order**	**Sequence homology (HK/RR, Identities/Identities)**	**Function prediction**
WP_011678182.1	WP_011678181.1	RH	YP_194286/YP_194287 44%/67% *L.aci*	Bile tolerance
WP_011677912.1	WP_003620636.1	RH	NP_814923/NP_814922 49%/75% *E.fae*	Vancomycin resistance
WP_011543754.1	WP_003613569.1	RH	WP_011374912.1/WP_011374913.1 42%/66% *L.sak*	Anaerobic regulation
WP_011678447.1	WP_003618182.1	HR	YP_194374/YP_194375 62%/91% *L.aci*	Protein hydrolysis, Acid resistance
WP_003621126.1	WP_003624707.1	HR	WP_011373988.1/WP_011373987.1 55%/82% *L.sak*	Vancomycin resistance, Anaerobic regulation
WP_011543855.1	WP_003620064.1	RH	WP_005726711.1/WP_060785076.1 95%/87% *L.cri*	Unknown
WP_011677872.1	WP_011677871.1	RH	WP_009557837.1/WP_009557836.1 83%/88% *L.equ*	Unknown

*L.aci, Lactobacillus acidophilus; Efae, Enterococcus faecalis; L.sak, Lactobacillus sakei; L.cri, Lactobacillus crispatus; L.equ, Lactobacillus equicursoris*.

Two histidine protein kinases, WP_011543855.1 and WP_011677872.1, had transmembrane regions (blue block), which can directly sense changes in the external environment. The PAS domain (PER-ARNTSIM domain) can sense changes of light, oxygen, redox potential, small molecule ligand and total cell energy, and is an important signal receptor domain (Furukawa-Hibi et al., 2015; Guo et al., 2015; Kasai et al., 2015). Compared with the structural regions of WP_011543855.1 and WP_011677872.1, those of WP_003620064.1 and WP_011677871.1 were relatively simple; both genes contained typical signal receiving domains (REC), and WP_011677871.1 contained a LytTR type signal output domain. In order to verify whether there is a correlation between the two TCS and cell autolysis, LBUL_RS04160 (coding gene of WP_011543855.1) and LBUL_RS00115 (coding gene of WP_011677872.1) were knocked out, respectively.

### Identification of the recombinant plasmids pUC19-HPK4160::EryBII and pUC19-HPK0115::EryBII

**Figure 6:**
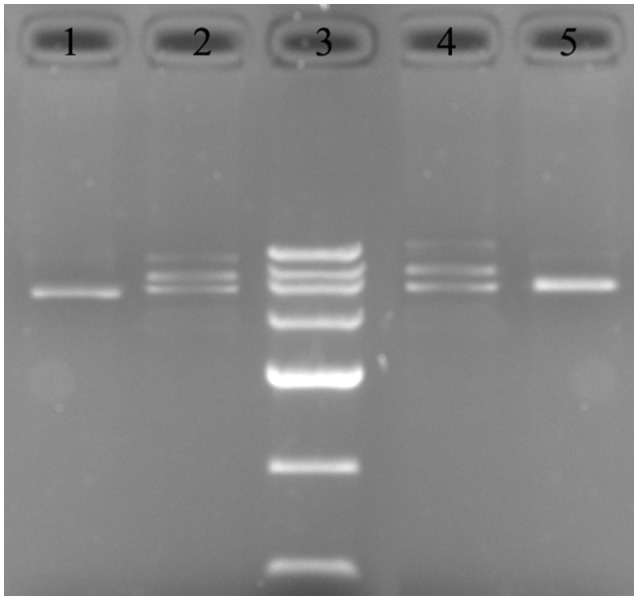
Recombinant plasmid mapping. 1: PCR detection of the HPK4160::EryBII gene from pUC19-HPK4160::EryBII using Primers 1 and 2; 2: pUC19-HPK4160::EryBII was cut by *Sph*I and *EcoR*I to 4.4, 2.7, 1.7 kbp fragments; 3: DNA marker III (200, 500, 800, 1,200, 2,000, 3,000, 4,500 bp); 4: pUC19-HPK0115::EryBII was cut by *Sph* I and *EcoR* I to 4.7, 2.7, 2.0 kbp fragments. 5: PCR detection of the HPK0115::EryBII gene from pUC19-HPK0115::EryBII using Primers 5 and 6.

The recombinant plasmids pUC19-HPK4160::EryBII obtained from transformed *E. coli* DH5α were submitted to digestion by *SphI* and *EcoR*I. Electrophoresis data indicated the presence of both the 4.4 kbp pUC19-HPK4160::EryBII and 1.7 kbp HPK4160::EryBII fragments, suggesting successful insertion of *EryB*II into pUC19-HPK4160. In agreement, PCR reactions using Primers 1 and 2 (Table 3) showed concordant data (Figure 6). Next, pUC19-HPK0115::EryBII identification was carried out in a similar way to pUC19-HPK4160::EryBII. After digestion, the 4.7 kb pUC19-HPK0115::EryBII and 2.0 kb HPK0115*::*EryBII fragments were all present, also suggesting successful HPK0115*::*EryBII insertion into the pUC19 vector. These findings were confirmed by PCR using Primers 5 and 6 (Figure 6).

**Table 3:** Primers used in this study.

**No**.	**Primer**	**Sequence(5′-3′)**	**Reference**
1	HPK4160-SphI-F	ACGCGCATGCCGCATGAACTTAAGACGCCC	This study
2	HPK4160-EcoRI-R	ACATGAATTCTTGCGGCTGTGGCTCTTATC	This study
3	EryB-BfaI-F	CCGCTAGATGACCACCGACGCCGCGACG	This study
4	EryB-BstEII-R	CGGGTAACCTCACTGCAACCAGGCTTCCGG	This study
5	HPK0115-SphI-F	ACGCGCATGCCGCGGGCAGGCAAAAAG	This study
6	HPK0115-EcoRI-R	ACATGAATTCAACGCAGCGGATGATGCTTA	This study
7	EryB-BstEII-F	CGGGTAACCATGACCACCGACGCCGCGACG	This study
8	EryB-BstYI-R	CGAGATCCTCACTGCAACCAGGCTTCCGG	This study
9	HPK0115-SalI-F	ACGCGTCGACATGATCAACAGCCTGTTC	This study
10	HPK0115-SphI-R	ACATGCATGCCTATCCCTTCTGAATAACT	This study
11	HATPase-NdeI-F	GGAATTCCATATGATGGTAAATATCGTAAGCATCA	This study
12	HATPase-BamHI-R	CGGGATCCCTATCCCTTCTGAATAACT	This study
13	RR0110-NdeI-F	GGAATTCCATATGATGCTAGCCATCATCATTT	This study
14	RR0110-BamHI-R	CGGGATCCTTAAACAAGGTCATTTT	This study

### Screening and identification of mutant strains

The recombinant plasmids pUC19-HPK4160::EryBII and pUC19-HPK0115::EryBII were transformed into *L. bulgaricus* ATCC BAA-365, respectively, by electroporation. After culture for 2 h at 37°C, the transformed bacteria were transferred on solid MRS medium with 0.5 M sucrose and erythromycin (50 μg/mL) for selection. Next, candidate colonies were plated onto MRS agar with ampicillin (50 μg/mL). The double-crossover mutant bacteria could not grow in the latter conditions. Finally, three mutants *L. bulgaricus* Δ*H4160* 1-3 with pUC19-HPK4160::EryBII and one double-crossover mutant *L. bulgaricus* Δ*H0115* 1 with pUC19-HPK0115:: EryBII were chosen in the second round.

**Figure 7:**
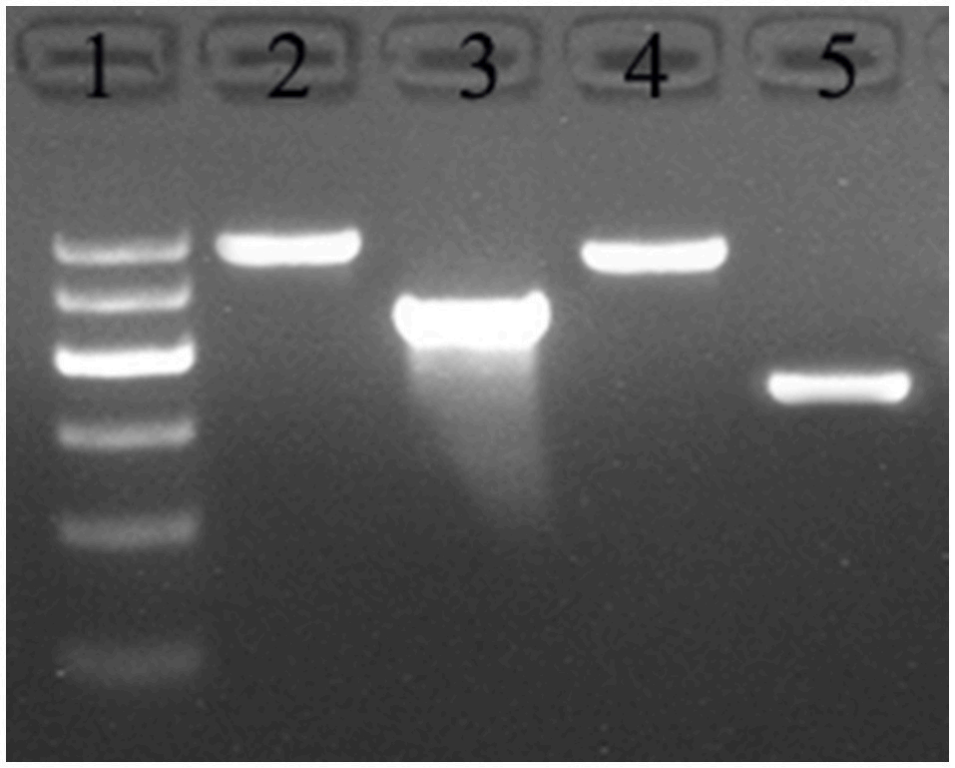
Detection of HPK gene knockout *L. bulgaricus*. 1: DNA marker D2000 (100, 250, 500, 750, 1,000, 2,000); 2: PCR with Primers 5 and 6 from mutant Δ*H0115*-1 genome; 3: PCR with Primers 5 and 6 from BAA-365 genome; 4: PCR with Primers 1 and 2 from mutant Δ*H4160*-1 genome; 5: PCR with Primers 1 and 2 from BAA-365 genome.

DNA was obtained from the *L. bulgaricus* ΔH4160-1 and wildtype BAA-365 genomes, after 24 h of culture in MRS broth, and amplified by PCR with Primers 1 and 2. This yielded 0.5 and 1.7 kb amplicons from wildtype and mutant genomic DNAs, respectively (Figure 7); the ~1.2 kb difference reflected the inserted erythromycin resistance gene. These data confirmed successful LBUL_RS04160 knockout by inserting the erythromycin resistance gene. The identification of LBUL_RS00115 mutant *L. bulgaricus* ΔH0115-1 was carried out as described above (Figure 7).

**Figure 8:**
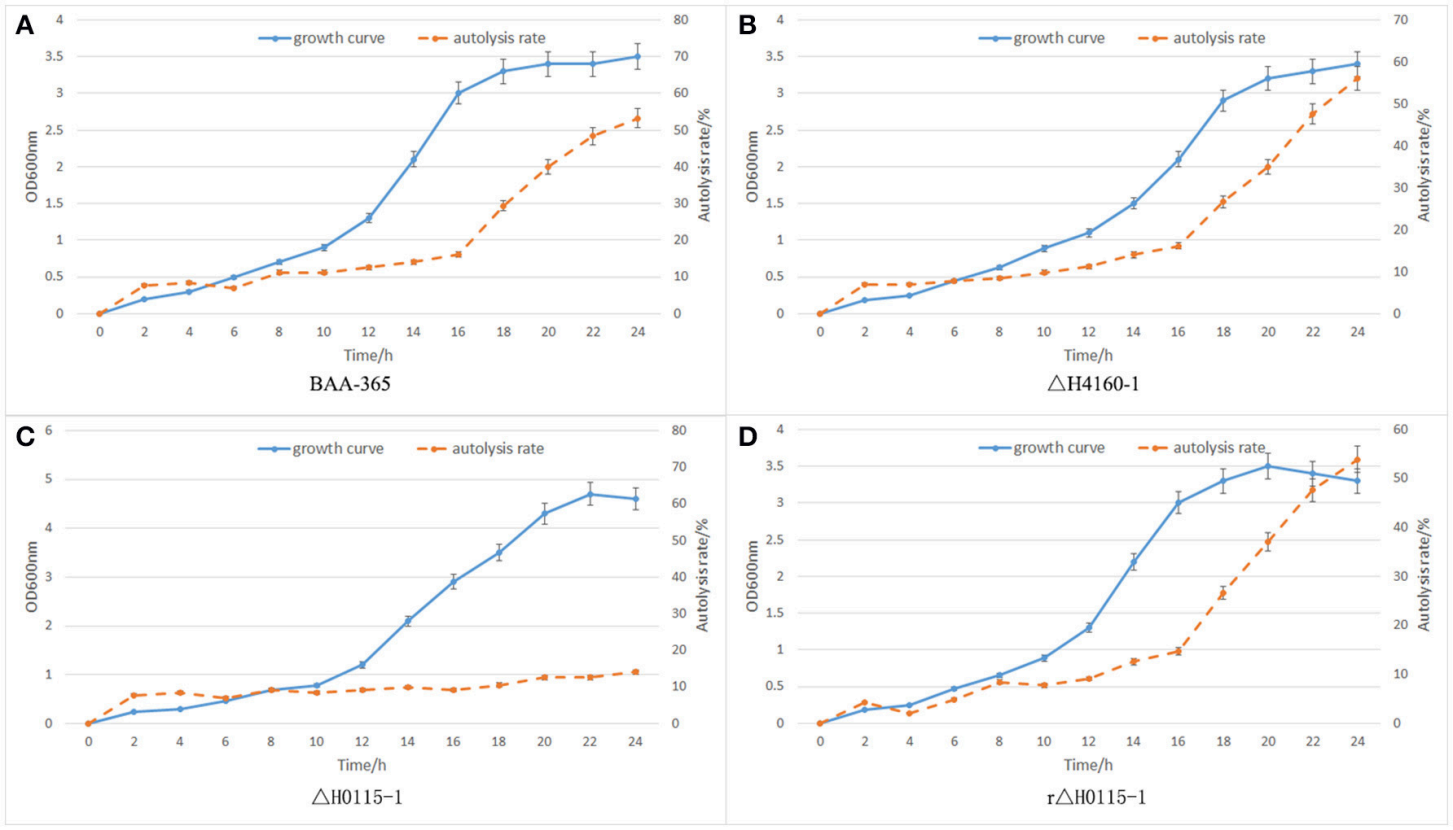
Autolysis data. **(A)** BAA-365; **(B)** ΔH4160-1; **(C)** ΔH0115-1; **(D)** rΔH0115-1.

For complementation, pMG56e-HPK0115 was transformed into *L. bulgaricus* Δ*H0115*-1 by electroporation. The *HPK0115*-complemented strain was named rΔH0115-1. As shown in Figure 8D, rΔH0115-1 autolysis was markedly increased in comparison with that of Δ*H0115*-1, with no significant difference compared with that of wild type. These findings suggested that *HPK0115* complementation restituted the autolytic capacity.

### Autolysis assessment data

**Figure 9:**
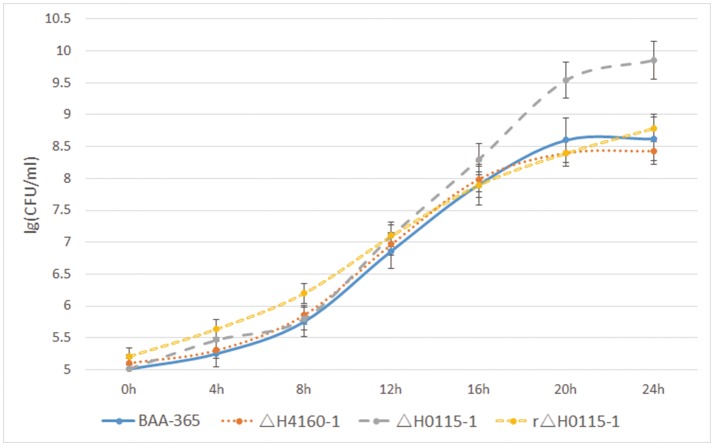
Results of colony counts (lg CFU/ml). The number of viable bacteria per milliliter of bacteria was measured by colony counts at different time points (0, 4, 8, 12, 16, 20, 24 h).

**Figure 10:**
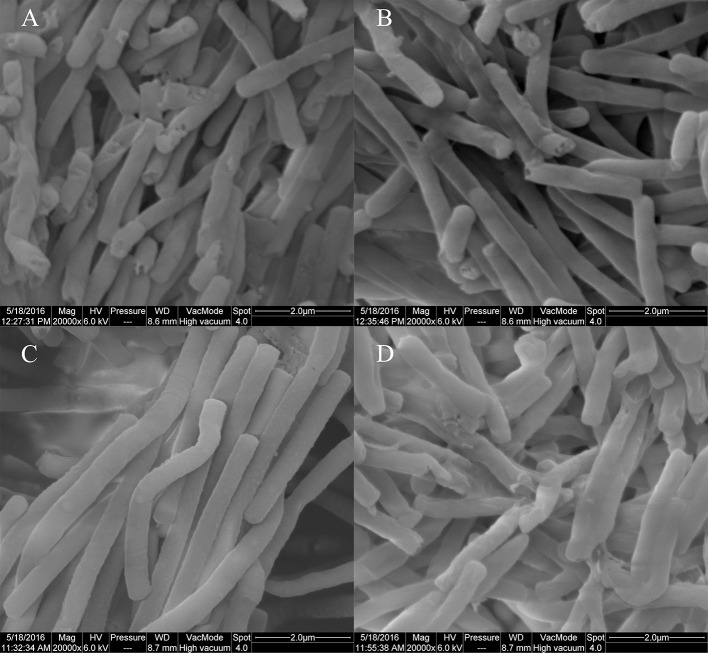
Results of electron microscopy (20000×). **(A)**:BAA-365; **(B)**:ΔH4160-1; **(C)**:ΔH0115-1; **(D)**: rΔH0115-1.

In BAA-365, ΔH4160-1, ΔH0115-1, and rΔH0115-1, autolysis monitoring results revealed no significant differences between the LBUL_RS04160 gene knockout strain ΔH4160-1 and wide type strain BAA-365; this indicated that LBUL_RS04160 gene was not associated with cell autolysis. However, autolysis rate of the LBUL_RS00115 gene knockout strain ΔH0115-1 was starkly reduced compared with the value obtained for the wild type strain at the 16 h time point. In addition, a markedly enhanced maximum OD value was obtained in the knockout mutant compared with wild type; this indicated that the density of *L. bulgaricus* population was significantly increased when the LBUL_RS00115 gene was knocked out (Figure 8). In order to further demonstrate that the autolysis of ΔH0115-1 changed significantly, we monitored the colony counts of the four strains at different time points, the results shows that when the bacteria grown to stationary phase, the viable count of ΔH0115-1 is significantly higher than that of other bacteria (Figure 9). The four bacteria grown to 24 h were observed by electron microscopy, it can be seen only ΔH0115-1 bacterial cell wall is still relatively complete, but the other three strains of cell wall can be seen obvious damage (Figure 10). These findings indicated a significant function for LBUL_RS00115 in *L. bulgaricus* autolysis. Meanwhile, a reduced autolysis remained in LBUL_RS00115 knockout organisms, implying the contribution of additional genes the autolytic process in *L. bulgaricus*.

### Two-hybrid analysis results between WP_011677872.1 and WP_011677871.1

**Figure 11:**
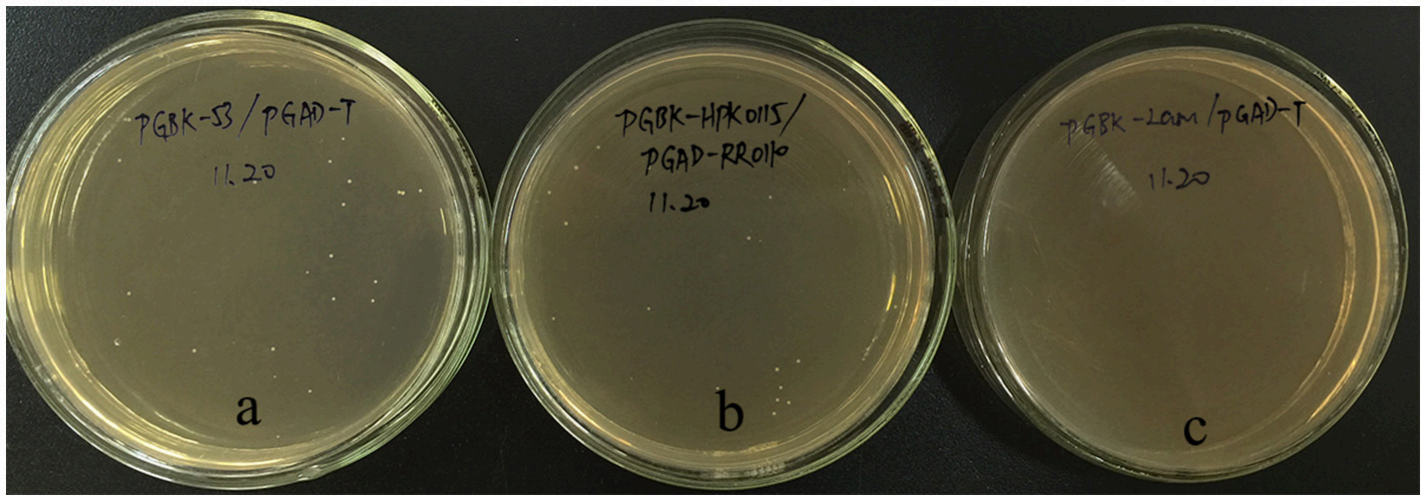
WP_011677872.1 and WP_011677871.1 interaction in a yeast two-hybrid assay. **(a)** pGBKT7-53/pGADT7-T; **(b)** pGBK-HPK0115/pGAD-RR0110; **(c)** pGBKT7-Lam/pGADT7-T.

The yeast two-hybrid system represents a well-known method in identifying protein interactions (Chini, 2014; Ferro et al., 2014). To determine whether the WP_011677872.1 (encoded by LBUL_RS00115) and WP_011677871.1 (encoded by LBUL_RS00110) proteins interact, two-hybrid system plasmids were generated with the HATPase-c domain gene of LBUL_RS00115 and full LBUL_RS00110 gene. The two-hybrid plasmids pGBK-HPK0115 and pGAD-RR0110 were cotransformed into *S. cerevisiae* Y_2_HGOLD. The cotransformants could grow on SD/-Trp-Leu-His plates with 0.1 mg·mL^−1^ aureobasidin A (Clontech) (Figure 11). These data demonstrated that these cotransformants activated both HIS3 and AUR1-C reporters, confirming interaction occurrence between WP_011677872.1 and WP_011677871.1.

## Discussion

The autolytic ability of LAB is essential for their use in food industry (Visweswaran et al., 2013). Previous research in our laboratory demonstrated that N-acetylmuramidase has a critical function in *L. bulgaricus* autolysis (Pang et al., 2014), as one of the major degraders of the cell wall. However, we are more interested in which protein transfer autolysis signals to N-acetylmuramidase. TCS are bacterial components that sense the surrounding environment (Marchadier and Hetherington, 2014; Yu et al., 2014), and LAB autolysis usually occurs at high cell density. It remains unclear whether there is a correlation between cell autolysis and TCS. In this study, the genes of two TCS whose functions are unknown were knocked out, respectively; results showed that autolysis rates were markedly lower for the LBUL_RS00115 gene mutant in comparison with the wild type strain BAA-365, which suggested that LBUL_RS00115 (coding gene of WP_011677872.1) contributes to *L. bulgaricus* autolysis. Furthermore, we found a direct interaction, including a phosphorelay, between WP_011677872.1 and WP_011677871.1 in this study. The interaction was characterized by yeast two-hybrid analysis. The above results demonstrated that the TCS WP_011677872.1/WP_011677871.1 is related to cell autolysis in *L. bulgaricus*, confirming our previous assumptions. However, whether the response regulator of this TCS can directly regulate the N-acetylmuramidase gene needs to be further investigated. The regulatory system of WP_011677872.1/WP_011677871.1 in *L. bulgaricus* could be a novel target for controlling cell autolysis. N-acetylmuramidase is involved in other metabolic processes *in vivo*, such as bacterial division, less impact on bacteria is produced by regulating TCS than N-acetylmuramidase. This study provides new insights for understanding autolysis regulation in *L. bulgaricus*.

## Author contributions

XP and JLv contributed in study conception and experimental design. YY and LL carried out vector construction experiments. SZ carried out Two-hybrid analysis experiments. XP and CM wrote the manuscript. PT and WG carried out the autolysis detection experiments. All authors have read and approved of the manuscript.

### Conflict of interest statement

The authors declare that the research was conducted in the absence of any commercial or financial relationships that could be construed as a potential conflict of interest.
